# Psychiatric disorders and cardiovascular diseases during the diagnostic workup of potential breast cancer: a population-based cohort study in Skåne, Sweden

**DOI:** 10.1186/s13058-019-1232-y

**Published:** 2019-12-10

**Authors:** Qing Shen, Anna Jöud, Maria E. C. Schelin, Arvid Sjölander, Yang Cao, Pär Sparén, Katja Fall, Kamila Czene, Unnur Valdimarsdóttir, Fang Fang

**Affiliations:** 1 Department of Medical Epidemiology and Biostatistics, Karolinska Institutet, Box 281, SE-171 77 Stockholm, Sweden; 2 Epidemiology and Register Centre South, Region Skåne, SE-221 85 Lund, Sweden; 3 Division of Occupational and Environmental Medicine, Department of Laboratory Medicine Lund, Lund University, SE-221 00 Lund, Sweden; 4 Clinical Epidemiology and Biostatistics, School of Medical Sciences, Örebro University, SE-701 82 Örebro, Sweden; 5 Department of Epidemiology, Harvard School of Public Health, Harvard University, Boston, MA 02115 USA; 6 Center of Public Health Sciences, Faculty of Medicine, University of Iceland, IS-101 Reykjavík, Iceland

**Keywords:** Breast cancer, Diagnostic workup, Psychiatric disorder, Cardiovascular disease

## Abstract

**Background:**

An increasing number of women are evaluated for potential breast cancer and may experience mental distress during evaluation. We aim to assess the risks of psychiatric disorders and cardiovascular diseases during the diagnostic workup of potential breast cancer.

**Methods:**

All women with a new diagnosis of unspecified lump in breast (*N* = 15,714), benign tumor or breast cancer in situ (*N* = 4435), or breast cancer (*N* = 8512) during 2005–2014 in Skåne, Sweden, were considered as exposed to a breast diagnostic workup. We used multivariable Poisson regression to compare rates of psychiatric disorders and cardiovascular diseases during the 6 weeks before the date of diagnosis of these women with the corresponding rates of women not undergoing such workup. The commonest waiting time for breast cancer patients was 6 weeks during the study period. A within-individual comparison was performed to control for potential unmeasured time-stationary confounders.

**Results:**

Compared to the reference, we found a higher rate of psychiatric disorders during the 6 weeks before diagnosis of benign tumor or breast cancer in situ (incidence rate ratio [IRR], 1.3; 95% confidence interval [CI], 1.1 to 1.5) and breast cancer (IRR, 1.4; 95% CI, 1.2 to 1.6). A higher rate was also noted for cardiovascular diseases (IRR, 1.3; 95% CI, 1.1 to 1.6 for benign tumor or breast cancer in situ, and IRR, 1.9; 95% CI, 1.8 to 2.0 for breast cancer). The rate increases for breast cancer were greater comparing a diagnostic workup due to symptoms to a workup due to screening. Little rate increase of neither psychiatric disorders nor cardiovascular diseases was noted during the 6 weeks before the diagnosis of unspecified lump in breast. The within-individual comparison largely confirmed these findings.

**Conclusions:**

Women with benign and malignant breast tumor had increased rates of psychiatric disorders and cardiovascular diseases during the waiting for a final diagnosis.

## Background

A growing proportion of the global population is currently being evaluated for potential cancer, due to population-based or opportunistic screening. A diagnostic workup of potential cancer—commonly defined as the period starting from the first suspicion of cancer until the final diagnosis or primary treatment—may, independently, have negative health implications. Present knowledge of such implications is mostly limited to cancer over-diagnosis and over-treatment [1]. Although accumulating evidence suggests that the diagnostic workup of cancer, regardless of the final result, affects the general health and well-being of the affected individuals [2–6], little attention has been devoted to assessing concrete and severe health outcomes in relation to cancer diagnostic workup.

Psychiatric disorders and acute cardiovascular events have been associated with stressful life events [7–13], including receiving a cancer diagnosis [14–16]. Our recent findings demonstrate that cancer patients have highly increased risks of psychiatric disorders and external injuries, not only after cancer diagnosis but also during the immediate time period before diagnosis [17, 18]. The increased risks before cancer diagnosis might be attributable to the onset of cancer symptoms but could also be due to the psychological distress experienced during the clinical evaluation of a potential cancer [17, 18]. To our best knowledge, no study has however specifically assessed the risks of psychiatric disorders and cardiovascular diseases during cancer diagnostic workup. Finally, the population currently being evaluated for potential cancer is growing, with or without an eventual cancer diagnosis [19]. It is important therefore to understand whether this population, especially the ones who are not eventually diagnosed with cancer, experiences a similar rise in the risk of these severe outcomes.

To this end, we performed a large population-based study in Sweden to assess the rates of psychiatric disorders and cardiovascular diseases during the diagnostic workup of potential breast cancer, due to either findings from screening or symptoms, with a focus on the waiting time before receiving a final diagnosis.

## Material and methods

### Study design

Skåne is a region in southern Sweden with a population size of ~ 1.3 million [20]. The Skåne Healthcare Register (SHR) includes all levels of health care provided in Skåne, including dates of healthcare visits and diagnostic and procedure codes (surgical or non-surgical). The coverage of SHR approaches 100% concerning both inpatient and outpatient specialist care and primary care. The Swedish Cancer Register has complete information on all newly diagnosed cancers in Sweden since 1958 [21]. Using the Total Population Register that includes information on birth, migration, and death of all Swedish residents, we conducted a cohort study including all adult women (≥ 18 years) residing during 2005–2014 in Skåne (*N* = 608,140). These women were individually followed from January 1, 2005, to a diagnosis of breast cancer, a diagnosis of any other cancer, total mastectomy, emigration out of Skåne, death, or December 31, 2014, whichever occurred first, through cross-linkages to the Cancer Register and the SHR, using the individually unique personal identification numbers.

Patients with a benign breast tumor undergo a similar diagnostic workup as breast cancer patients, whereas unspecified lump in breast is the most common symptom for symptomatic breast cancer and the main reason for symptom-based breast diagnostic workup [22]. We therefore first identified all women that received a diagnosis of breast cancer during the study period according to the Cancer Register (*N* = 8512). Among women without a breast cancer diagnosis, we then identified women with benign tumor in breast or breast cancer in situ according to the Cancer Register and SHR (*N* = 4435). As vast majority of women with benign tumor in breast or breast cancer in situ have a benign tumor (98.7%), we refer to this group as patients with benign tumor. Finally, among women with neither breast cancer nor benign tumor or breast cancer in situ, we identified women with unspecified lump in breast according to SHR (*N* = 15,714). All these women were defined as exposed to a diagnostic workup of potential breast cancer (Additional file 1: Figure S1). For women with breast cancer, we used the date of diagnosis as registered in the Cancer Register (95%) or the SHR (5%), whichever ever occurred first. For patients with unspecified lump and benign tumor, we used the date of last corresponding diagnosis during a workup from the SHR as the date of diagnosis, if multiple diagnoses were identified. The International Classification of Diseases (ICD) codes used for different breast diagnoses are listed in Additional file 1: Table S1.

According to the national cancer care program in Sweden, the median waiting time from a well-founded suspicion of breast cancer to the start of initial cancer treatment is expected to be 28 days, and the waiting time was 6 weeks for 80% of the breast cancer patients in Skåne [23]. In the present study, the number of healthcare visits started to increase from the 6th week before diagnosis, compared to earlier, among women that received a diagnosis of breast cancer during the follow-up (Additional file 1: Figure S2). We estimated therefore the rates of psychiatric disorders and cardiovascular diseases during the 6 weeks before the diagnosis of women that had a breast diagnostic workup. As a contrast, we also estimated the rates of psychiatric disorders and cardiovascular diseases during the time interval from diagnosis to the day before surgery, among women that received surgical treatment after diagnosis.

Women that did not have any breast diagnostic workup during follow-up contributed all their person-time to the reference group. Women that underwent a breast diagnostic workup also contributed person-time to the reference group before the start of such workup.

### Ascertainment of psychiatric disorders and cardiovascular diseases

Psychiatric disorders and cardiovascular diseases diagnosed through either primary or specialist inpatient or outpatient care, according to SHR, were used as the primary outcomes of interest. We used ICD-10 codes F10-F99 to identify all diagnoses of psychiatric disorders and I00-I99 to identify all diagnoses of cardiovascular diseases. Repeated events were taken into account apart from events that had the same diagnosis and occurred within 28 days of each other. Because there are deaths from cardiovascular diseases that are outside the hospital, we also identified deaths due to cardiovascular diseases that were not preceded by a related hospital visit from the Causes of Death Register and considered them as additional cardiovascular events. We classified psychiatric disorders as stress reaction or adjustment disorder, depression, anxiety, substance abuse, and others, and classified cardiovascular diseases as myocardial infarction, hypertensive diseases or aneurysm of the heart, embolism or thrombosis, stroke, and other diseases of the circulatory system. Corresponding ICD codes are listed in Additional file 1: Table S1.

### Statistical analysis

We described the characteristics of the exposed women according to their final diagnosis, including age at diagnosis, tumor stage (breast cancer patients alone), surgical treatment, cohabitation status, preexisting psychiatric disorders, and preexisting cardiovascular diseases. We estimated the crude incidence rates (IRs) of psychiatric disorders and cardiovascular diseases during the 6 weeks before diagnosis for all exposed women and during the waiting time for surgical treatment for women that received surgery after diagnosis, by dividing the number of events with the accumulated person-months at risk. We used Poisson regression to estimate the incidence rate ratios (IRRs) and their 95% confidence intervals (CIs), by comparing the IRs of different exposed women with the IRs of the reference group. Because we took into account repeated outcomes, we used a clustered sandwich estimator to account for the intra-individual correlation. We used attained age as the underlying timescale, and all analyses were adjusted for cohabitation status and registered parish (as a proxy for socioeconomic status), both ascertained from the Total Population Register, and preexisting psychiatric disorders or cardiovascular diseases. We ascertained preexisting psychiatric disorders or cardiovascular diseases from the SHR from 2004 onward and updated such information at the beginning of each time period (i.e., these variables were treated as time-varying).

To assess potential effect modifications, we performed stratified analyses by age, calendar period, cohabitation status, and preexisting psychiatric disorders or cardiovascular diseases and used the Wald test to assess the differences between the stratum-specific estimates. We separately analyzed psychiatric disorders and cardiovascular diseases that were identified from primary care alone and from specialist care alone, to examine whether the results would differ by type of healthcare level used to ascertain the studied outcomes. To eliminate potential confounding due to time-stationary factors, we additionally performed a within-individual comparison using conditional Poisson regression [24]. In this analysis, we compared the IRs of psychiatric disorders and cardiovascular diseases during the 6 weeks before diagnosis with the corresponding IRs during the period before these 6 weeks for each woman that had a breast diagnostic workup. Finally, to assess whether the results would differ for a breast diagnostic workup due to symptoms compared to a workup due to screening findings, we performed a separate analysis during 2009–2014 for which information on breast cancer screening was largely available in SHR. Because we used psychiatric disorders and cardiovascular diseases as two proxies for severe health outcomes related to stress, in a sensitivity analysis of the entire study sample, we also estimated the IRR of having either a psychiatric disorder or a cardiovascular disease during the 6 weeks before diagnosis among women with different breast diagnoses.

The assumption of equal-dispersion for Poisson regression was found to hold for all analyses. All analyses were performed in SAS 9.4 (SAS Institute) and STATA 13.1 (StataCorp LP). The study was approved by the Central Ethical Review Board in Stockholm, Sweden.

## Results

The mean age at diagnosis was 41 years for women with unspecified lump in breast, 45 years for women with benign tumor, and 63 years for women with breast cancer (Table 1). Ninety percent of breast cancer patients received surgical treatment, whereas 30% of patients with benign tumor and < 2% of patients with unspecified lump in breast received surgical treatment. Almost half of the breast cancer patients diagnosed during 2009–2014 had a diagnostic workup due to screening (Additional file 1: Table S2).

**Table 1:** Characteristics of women without a breast diagnostic workup and women with a breast diagnostic workup by their final diagnosis, a population-based cohort study during 2005–2014 in Skåne, Sweden

Characteristics	Women without breast diagnostic workup*	Women with lump in breast	Women with benign tumor	Women with breast cancer
No. of women	579,447	15,714	4435	8512
Age in years at diagnosis, mean (SD)	–	41 (15)	45 (16)	63 (14)
Tumor stage				
T0N0M0/stage 0/ stage I	–	–	–	4456 (52.3%)
Stage II	–	–	–	3109 (36.5%)
Stages III and IV	–	–	–	670 (7.9%)
Missing stage	–	–	–	277 (3.3%)
Surgical treatment				
Yes	–	288 (1.8%)	1339 (30.2%)	7662 (90.0%)
No	–	15,426 (98.2%)	3096 (69.8%)	850 (10.0%)
Cohabitation status				
Cohabitating	365,287 (63.0%)	7304 (46.5%)	2105 (47.5%)	4400 (51.7%)
Non-cohabitating	214,160 (37.0%)	8410 (53.5%)	2330 (52.5%)	4112 (48.3%)
Preexisting psychiatric disorders				
Yes	43,380 (7.5%)	4426 (28.2%)	1037 (23.4%)	1890 (22.2%)
No	536,067 (92.5%)	11,288 (71.8%)	3398 (76.6%)	6622 (77.8%)
Preexisting cardiovascular diseases				
Yes	47,362 (8.2%)	2980 (19.0%)	850 (19.2%)	3189 (37.5%)
No	532,085 (91.8%)	12,734 (81.0%)	3585 (80.8%)	5323 (62.5%)

*SD* standard deviation

*32 women had mastectomy before the start of follow-up and was not included in further analyses, leaving 579,447 women without any diagnostic workup

### Psychiatric disorders

There were 521 women with unspecified lump in breast (3.3% of all women with lump), 145 women with benign tumor (3.3% of all women with benign tumor), and 253 women with breast cancer (3.0% of all women with breast cancer) that were diagnosed with psychiatric disorders during the 6 weeks before diagnosis. Compared with the reference group, we found an increased rate of psychiatric disorders during the 6 weeks before diagnoses among all exposed women (IR, 25.5 per 1000 person-months; IRR, 1.2; 95% CI, 1.1 to 1.3). The increased rate was mainly noted among women with benign tumor (IRR, 1.3; 95% CI, 1.1 to 1.5) or breast cancer (IRR, 1.4; 95% CI, 1.2 to 1.6) although only marginally among women with unspecified lump in breast (IRR, 1.1; 95% CI, 1.0 to 1.2) (Table 2). Among women with breast cancer, the rate increase was greater for a higher stage compared to a lower stage cancer. A statistically significant rate increase was noted for all individual psychiatric disorders studied before diagnosis of breast cancer, but only for stress reaction or adjustment disorder and substance abuse before diagnosis of benign tumor and for anxiety before diagnosis of unspecified lump in breast (Table 3).

**Table 2:** Incidence rates (IRs, per 1000 person-months) and incidence rate ratios (IRRs) of psychiatric disorders and cardiovascular diseases during the 6 weeks before diagnosis of women that underwent a breast diagnostic workup, a population-based cohort study during 2005–2014 in Skåne, Sweden

	No. of events	Crude IR	IRR (95% CI)^†^
Psychiatric disorder			
Reference group*	954,351	17.6	1.0
Unspecified lump in breast	578	26.4	1.1 (1.0–1.2)
Benign tumor	155	25.2	1.3 (1.1–1.5)
Breast cancer	278	24.0	1.4 (1.2–1.6)
T0N0M0/stage 0/stage I	149	24.5	1.4 (1.1–1.6)
Stage II	95	22.5	1.4 (1.1–1.7)
Stages III and IV	29	31.8	1.8 (1.2–2.6)
Missing stage	5	13.3	1.0 (0.4–2.5)
Cardiovascular disease			
Reference group*	1,094,327	20.1	1.0
Unspecified lump in breast	297	13.6	1.1 (1.0–1.2)
Benign tumor	116	18.9	1.3 (1.1–1.6)
Breast cancer	777	67.2	1.9 (1.8–2.0)
T0N0M0/stage 0/stage I	334	55.0	1.7 (1.5–1.9)
Stage II	284	67.4	1.8 (1.6–2.1)
Stages III and IV	115	126.5	2.8 (2.4–3.3)
Missing stage	44	118.0	2.9 (2.1–3.9)

*IR* incidence rate, *IRR* incidence rate ratio

*Reference group included person-time accumulated from women who did not have any breast diagnostic workup during the follow-up and the person-time accumulated before the start of workup from women with a breast diagnostic workup during the follow-up

^†^*CI* confidence interval. IRRs were estimated using Poisson regression, by using attained age as the underlying timescale and adjusting for cohabitation status, registered parish as a proxy for socioeconomic status, and preexisting psychiatric disorders (for outcome of psychiatric disorders) or preexisting cardiovascular diseases (for outcome of cardiovascular diseases)

**Table 3:** Incidence rates (IRs, per 1000 person-months) and incidence rate ratios (IRRs) of individual psychiatric disorders and cardiovascular diseases during the 6 weeks before diagnosis of women that had a breast diagnostic workup, a population-based cohort study during 2005–2014 in Skåne, Sweden

	Reference group*			Unspecified lump in breast			Benign tumor			Breast cancer		
	No.	Crude IR	IRR (95% CI)^†^	No.	Crude IR	IRR (95% CI)^†^	No.	Crude IR	IRR (95% CI)^†^	No.	Crude IR	IRR (95% CI)^†^
Psychiatric disorder												
Stress reaction or adjustment disorder	148,638	2.7	1.0	102	4.7	1.2 (1.0–1.5)	32	5.2	1.6 (1.1–2.3)	38	3.3	1.5 (1.1–2.0)
Depression	296,613	5.5	1.0	165	7.5	1.1 (0.9–1.2)	47	7.6	1.2 (0.9–1.7)	91	7.9	1.3 (1.1–1.6)
Anxiety	198,330	3.6	1.0	147	6.7	1.3 (1.1–1.6)	31	5.0	1.2 (0.8–1.7)	51	4.4	1.3 (1.0–1.7)
Substance abuse	58,007	1.1	1.0	31	1.4	1.1 (0.8–1.6)	16	2.6	2.3 (1.4–3.7)	24	2.1	1.8 (1.2–2.7)
Others	252,763	4.7	1.0	133	6.1	1.0 (0.8–1.2)	29	4.7	0.9 (0.6–1.3)	74	6.4	1.4 (1.1–1.7)
Cardiovascular disease												
Myocardial infarction	18,727	0.3	1.0	3	0.1	0.9 (0.3–2.8)	2	0.3	1.8 (0.4–7.1)	6	0.5	0.8 (0.4–1.9)
Hypertensive diseases or aneurysm of the heart	525,505	9.7	1.0	130	5.9	0.9 (0.8–1.1)	63	10.2	1.5 (1.1–1.9)	419	36.2	2.1 (2.0–2.4)
Embolism or thrombosis	13,511	0.2	1.0	3	0.1	0.8 (0.2–2.4)	1	0.2	0.8 (0.1–5.9)	16	1.4	3.4 (2.1–5.5)
Stroke	33,808	0.6	1.0	3	0.1	0.4 (0.1–1.3)	1	0.2	0.4 (0.1–3.0)	20	1.7	1.5 (1.0–2.4)
Other diseases of the circulatory system	502,776	9.3	1.0	158	7.2	1.3 (1.1–1.5)	49	8.0	1.2 (0.9–1.6)	316	27.3	1.7 (1.5–1.9)

*IR* incidence rate, *IRR* incidence rate ratio

*Reference group included person-time accumulated from women who did not have any breast diagnostic workup during the follow-up and the person-time accumulated before the start of workup from women with a breast diagnostic workup during the follow-up

^†^*CI* confidence interval. IRRs were estimated using Poisson regression, by using attained age as the underlying timescale and adjusting for cohabitation status, registered parish as a proxy for socioeconomic status, and preexisting psychiatric disorders (for outcome of psychiatric disorders) or preexisting cardiovascular diseases (for outcome of cardiovascular diseases)

The rate increase of psychiatric disorders during the 6 weeks before diagnosis was greater among women diagnosed in early calendar period or women without preexisting psychiatric disorders, compared to women diagnosed in late calendar period or women with a history of psychiatric disorders (*P* for differences < 0.05) (Additional file 1: Table S3). Apart from unspecified lump in breast, the rate increase was noted both for psychiatric disorders ascertained from primary care alone and for those ascertained from specialist care alone (Additional file 1: Figure S3). We also noted an increased rate of psychiatric disorders during the waiting time for surgical treatment among women that received surgical treatment after diagnosis of breast cancer, but not after diagnosis of benign tumor or unspecified lump (Additional file 1: Table S4).

The within-individual comparison confirmed the findings of the main analyses (Table 4). When investigating the mode of detection (data restricted to 2009–2014), we found a statistically significantly increased rate of psychiatric disorders during the 6 weeks before diagnosis of benign tumor and breast cancer for women that underwent a diagnostic workup due to symptoms but not screening (Table 5).

**Table 4:** Incidence rates (IRs, per 1000 person-months) and incidence rate ratios (IRRs) of psychiatric disorders and cardiovascular diseases during 6 weeks before diagnosis among women that had a breast diagnostic workup during 2005–2014 in Skåne, Sweden, a within-individual comparison

	No. of events	Crude IR	IRR (95% CI)^†^
Psychiatric disorder			
Reference period*	29,010	17.5	1.0
Unspecified lump in breast	578	26.4	1.0 (0.9–1.1)
Benign tumor	155	25.2	1.3 (1.1–1.5)
Breast cancer	278	24.0	1.4 (1.2–1.6)
Cardiovascular disease			
Reference period*	25,675	15.5	1.0
Unspecified lump in breast	297	13.6	0.9 (0.8–1.1)
Benign tumor	116	18.9	1.3 (1.1–1.5)
Breast cancer	777	67.2	1.8 (1.7–2.0)

*IR* incidence rate, *IRR* incidence rate ratio

*Reference period included person-time accumulated before the start of workup from women with a breast diagnostic workup during the follow-up

^†^*CI* confidence interval. IRRs were estimated using conditional Poisson regression, by using attained age as the underlying timescale

**Table 5:** Incidence rates (IRs, per 1000 person-months) and incidence rate ratios (IRRs) of psychiatric disorders and cardiovascular diseases during the 6 weeks before diagnosis, among women that had a breast diagnostic workup during 2009–2014 in Skåne, Sweden, analysis according to reason for diagnostic workup

	No. of events	Crude IR	IRR (95% CI)^†^
Psychiatric disorder			
Reference group*	679,051	20.8	1.0
Due to screening			
Unspecified lump in breast	27	27.7	1.2 (0.8–1.8)
Benign tumor	31	23.5	1.2 (0.8–1.7)
Breast cancer	69	20.6	1.0 (0.8–1.3)
Due to symptom			
Unspecified lump in breast	413	29.9	1.1 (1.0–1.3)
Benign tumor	78	31.7	1.3 (1.0–1.6)
Breast cancer	139	35.8	1.7 (1.4–2.0)
Cardiovascular disease			
Reference group*	740,161	22.7	1.0
Due to screening			
Unspecified lump in breast	22	22.6	1.3 (0.9–1.9)
Benign tumor	38	28.8	1.4 (1.1–2.0)
Breast cancer	131	39.1	1.3 (1.1–1.6)
Due to symptom			
Unspecified lump in breast	181	13.1	1.0 (0.9–1.1)
Benign tumor	44	17.9	1.2 (0.9–1.6)
Breast cancer	439	113.2	2.3 (2.1–2.5)

*IR* incidence rate, *IRR* incidence rate ratio

*Reference group included person-time accumulated from women who did not have any breast diagnostic workup during the follow-up and the person-time accumulated before the start of workup from women with a breast diagnostic workup during the follow-up

^†^*CI* confidence interval. IRRs were estimated using Poisson regression, by using attained age as the underlying timescale and adjusting for cohabitation status, registered parish as a proxy for socioeconomic status, and preexisting psychiatric disorders (for outcome of psychiatric disorders) or preexisting cardiovascular diseases (for outcome of cardiovascular diseases)

### Cardiovascular diseases

There were 284 women with unspecified lump in breast (1.8% of all women with lump), 110 women with benign tumor (2.5% of all women with benign tumor), and 708 women with breast cancer (8.3% of all women with breast cancer) that had a diagnosis of cardiovascular diseases during the 6 weeks before diagnosis. Compared with the reference group, we found an increased rate of cardiovascular diseases during the 6 weeks before diagnoses among all exposed women (IR, 30.0 per 1000 person-months; IRR, 1.5; 95% CI, 1.5 to 1.6). The increased rate was primarily noted among women with benign tumor (IRR, 1.3; 95% CI, 1.1 to 1.6) or breast cancer (IRR, 1.9; 95% CI, 1.8 to 2.0) but only marginally among women with unspecified lump in breast (IRR, 1.1; 95% CI, 1.0 to 1.2) (Table 2). The rate increase was greater for advanced cancer compared to a lower stage cancer among women with breast cancer. An increased rate was noted for most of the individual cardiovascular diseases studied before the diagnosis of breast cancer, whereas for hypertensive diseases or aneurysm of the heart before the diagnosis of benign tumor, and for other diseases of the circulation system before the diagnosis of unspecified lump in breast (Table 3).

A statistically significant rate increase of cardiovascular diseases was noted during the 6 weeks before diagnosis of breast cancer, regardless of age, calendar period, cohabitation status, or preexisting cardiovascular disease (Additional file 1: Table S3). However, among women with unspecified lump in breast, the IRR was statistically stronger during 2005–2009, compared to 2010–2014. Also, among women with benign tumor or breast cancer, the IRR was statistically significantly greater when the women were non-cohabitating, compared to women that were cohabitating (*P* for differences < 0.05). The results did not differ clearly by whether the outcomes were ascertained through primary care or specialist care alone (Additional file 1: Figure S3). A rate increase was also noted during the waiting time for surgical treatment among women with breast cancer, but not benign tumor or unspecified lump in breast (Additional file 1: Table S4).

The within-individual comparison largely confirmed these findings (Table 4). We found increased rate of cardiovascular diseases during the 6 weeks before diagnosis of benign tumor and breast cancer among women that had a breast diagnostic workup due to screening, and of breast cancer among women that had a diagnostic workup due to symptoms, when restricting the analysis to 2009–2014 (Table 5).

There were 688 women with unspecified lump in breast (4.4% of all women with lump), 227 women with benign tumor (5.1% of all women with benign tumor), and 754 women with breast cancer (8.9% of all women with breast cancer) that had either a psychiatric disorder diagnosis or a cardiovascular disease diagnosis during the 6 weeks before diagnosis. The corresponding IRRs (95% CIs) were 1.1 (1.1–1.2) for women with unspecified lump, 1.3 (1.1–1.4) for women with benign tumor, and 1.7 (1.6–1.8) for women with breast cancer during the 6 weeks before diagnosis.

## Discussion

This study is the first systematic assessment of clinically confirmed psychiatric disorders and cardiovascular diseases during a diagnostic workup of potential breast cancer, focusing on the time period before diagnosis. We found that women evaluated for a suspected breast cancer had increased rates of psychiatric disorders and cardiovascular diseases while waiting for their diagnosis. The rate increases were noted for women diagnosed with benign as well as malignant breast tumor, but minimally among women diagnosed with unspecified lump in breast. These rate increases appeared stronger for a diagnostic workup of breast cancer due to symptoms compared to a diagnostic workup due to screening findings, as well as among non-cohabitating women and women without previous history of psychiatric disorders.

Apart from over-diagnosis and over-treatment, other health impacts of cancer diagnostic workup remain largely unknown. The limited literature so far has focused on *self-reported* psychological symptoms and quality of life during cancer screening programs [25, 26] and on patients already diagnosed with cancer [2–6], whereas data are scarce on concrete and severe health outcomes during clinical evaluations for potential cancer, which could be due to either screening findings or symptoms. Specifically, little is known about such health impact among individuals that are evaluated for, but never diagnosed with, cancer. Diagnostic workup of potential cancer has been suggested to affect general health, especially the psychosocial spheres of function, including distress, fear and worry, anxiety, mood changes, sense of uncertainty, behavioral symptoms, concerns about future reproduction, and suicide [3–6]. Because almost all studies used self-reported quantitative measurements [3, 6, 27], the defining and measuring of immediate and potentially severe health outcomes of cancer diagnostic workup needs to be improved [25]. The generally low response rate and the highly selected study participants—mostly healthier than average—in cancer screening programs further underline the potential lack of generalizability of previous findings. Finally, the precise size of the population “exposed” to cancer diagnostic workup is unknown but presumably large, suggesting that cancer diagnostic workup and its resultant health impact do not only apply to cancer patients but also to a much larger population not destined to developing cancer.

Studies on cancer diagnostic workup are challenging because there is usually a lack of detailed data on the entire care process of patients with a suspected cancer. For example, it is difficult to follow patients over time and between various healthcare levels. Given the data sources concerning all health care provided to the entire female population of Skåne region, we had the unique opportunity to systematically assess the potential negative health impact of cancer diagnostic workup. Our novel results demonstrated increased risks of two concrete health outcomes that are known to be associated with severe psychological distress, namely psychiatric disorders and cardiovascular diseases, in relation to a diagnostic workup of potential breast cancer. The increased rates of these two outcomes during the 6 weeks before the diagnosis of breast cancer and benign breast tumor but not unspecified lump in breast might be due to severer symptoms and their resultant psychological distress among women with a benign tumor or cancer, compared to women with an unspecified lump. For instance, it has been hypothesized that the tumor-related inflammation may also contribute to the development of ill mental-health, including stress-related disorders [28, 29]. The fact that greater rate increase of psychiatric disorders and cardiovascular diseases was noted during a breast diagnostic workup due to symptoms, compared to mammography screening, might suggest that findings generated from previous studies using cancer screening programs might have underestimated the health impact of symptom-based clinical evaluations for potential cancer [30, 31]. Among all women that underwent a breast diagnostic workup in the present study, only 30% were eventually diagnosed with and treated for breast cancer. Finally, although no overall rate increase was observed among women with unspecified lump in breast, the present study did suggest a higher rate of psychiatric disorders in relation to the diagnostic workup of unspecified lump among women that were at low baseline risk of the outcome otherwise (i.e., without previous history of psychiatric disorders). The greater risk increase for women without a preexisting psychiatric disorder was also seen for women with benign tumor or breast cancer, highlighting again the impact of breast cancer diagnostic workup among the women with otherwise low risk of psychiatric disorders.

To our knowledge, we are the first to contrast the rates of psychiatric disorders and cardiovascular diseases during the waiting time for a potential cancer diagnosis to the corresponding rates during the waiting time for primary treatment. We focused on surgery, because 90% of the breast cancer patients received surgical treatment in the study. The rate increases of psychiatric disorders and cardiovascular diseases were similar during the 6 weeks before diagnosis compared to the rate increases during the time from diagnosis to surgery, suggesting a sustained high level of psychological distress both while awaiting for a diagnosis and after receiving such diagnosis. A limitation of the present study is that we had little clinical information about the women that underwent a breast diagnostic workup, except the estimated indication for workup, i.e., due to symptoms or screening. Future studies with more detailed clinical information are needed to better identify women at high risk of either psychiatric disorders or cardiovascular diseases during a breast diagnostic workup. Such additional knowledge, together with our findings, would better motivate clinical surveillance and, potentially, intervention of psychiatric disorders and cardiovascular diseases among high-risk women, both during the clinical evaluation of a potential breast cancer and when the patients are awaiting for the start of primary treatment.

Major strengths of our study include the large-scale population-based cohort design and the minimized selection and information biases given the prospectively and independently collected data from different registers and the complete follow-up. The coverage of the SHR on all levels of healthcare visits in Skåne made it possible to capture both severer outcomes that require specialist care and milder outcomes that require only primary care. The within-individual comparison, comparing different time periods of the same individual, eliminated further the potential impact of unknown and unmeasured confounders that are constant within each individual. Another novelty of the present study, compared to previous studies including our own [17], is the focus on the cancer diagnostic workup, especially the 6 weeks before diagnosis, to assess the role of clinical evaluation for a potential breast cancer on the risks of psychiatric disorders and cardiovascular disease, and to study not only women that were eventually diagnosed with breast cancer but also women that did not receive a cancer diagnosis after the clinical evaluation.

A limitation is the lack of data on other factors that might either confound or modify the association of cancer diagnostic workup with the risk of psychiatric disorders or cardiovascular diseases, including race or ethnicity, use of psychiatric medications, and family history of cancer, psychiatric disorders, or cardiovascular diseases. Further, the use of registered parish as a proxy for socioeconomic status and the use of cohabitation status as a proxy for marital status might have induced residual confounding to some extent. It also prohibited us from studying specific groups of vulnerable women, for instance, divorced or widowed women. Another limitation is the small number of outcomes in some subgroup analyses, which made the interpretation of these analyses difficult. Although some women had both psychiatric disorders and cardiovascular diseases during the 6 weeks before diagnosis, the proportions are small (0.7% of all women with lump, 0.09% of all women with benign tumor, and 0.8% of all women with breast cancer). We did not validate the diagnoses of the studied psychiatric disorders and cardiovascular diseases using SHR in the present study because previous studies have however shown satisfactory quality of such register-based definitions [32–34]. In addition, because of the increasing awareness and therefore registration during the study period in Sweden and Skåne, the definitions of psychiatric disorders and cardiovascular diseases included more milder conditions in later years compared to earlier years. Assuming that the psychological distress experienced during a breast diagnostic workup is related to relatively severe forms of these outcomes, such a temporal trend might have contributed to the stronger association noted during the earlier calendar period, compared to later calendar period. Another possible contribution to the stronger association during earlier calendar period is the increasing uptake of breast screening program during the study period, as we found that the association was indeed stronger for breast diagnostic workup due to symptoms, compared to diagnostic workup due to screening. Furthermore, we could not identify the precise time when the patient was informed about cancer diagnosis, if there was a cancer diagnosis, using either the Cancer Register or SHR. We focused on the diagnostic workup of breast cancer in the present study, because patients with breast cancer have been reported to be at the highest prevalence of psychiatric disorders, compared to patients with other cancer types [35]. The findings of the present study might also be generalizable to diagnostic workup of other cancer types. For instance, increased level of psychological distress has been reported among men undergoing a clinical evaluation for potential prostate cancer, especially when waiting for the result of prostate biopsy [6]. Finally, whether or not the present findings are generalizable to other populations remains to be assessed.

## Conclusions

In conclusion, using a large population-based cohort with a follow-up of 10 years, we demonstrated that women had increased risks of psychiatric disorders and cardiovascular diseases during diagnostic workup of potential breast cancer. The risk increases were greater when the diagnostic workup was due to symptoms, compared to when the diagnostic workup was due to screening. Future research is needed to understand the underlying reasons for such risk increases.

## Supplementary information

**Additional file 1:** **Table S1.** ICD codes for breast disorders, psychiatric disorders, and cardiovascular diseases. **Figure S1.** Study flow chart, a population-based cohort study during 2005-2014 in Skåne, Sweden. **Figure S2.** Weekly frequency of healthcare visits before and after the date of diagnosis from women with breast cancer, a population-based cohort study during 2005-2014 in Skåne, Sweden. **Table S2.** Characteristics of women with a breast diagnostic workup by their final diagnosis, a population-based cohort study during 2009-2014 in Skåne, Sweden. **Table S3.** Incidence rates (IRs, per 1000 person-months) and incidence rate ratios (IRRs) of psychiatric disorders and cardiovascular diseases during the six weeks before diagnosis of women that had a breast diagnostic workup, according to age, calendar period, cohabitation status, and preexisting psychiatric disorder or cardiovascular disease, a population-based cohort study during 2005-2014 in Skåne, Sweden. **Figure S3.** Incidence rate ratios and their 95% confidence intervals of psychiatric disorders and cardiovascular diseases during the six weeks before diagnosis of women that had a breast diagnostic workup, by type of healthcare visit, a population-based cohort study during 2005-2014 in Skåne, Sweden.*. **Table S4.** Incidence rates (IRs, per 1000 person-months) and incidence rate ratios (IRRs) of psychiatric disorders and cardiovascular diseases during the waiting time for surgical treatment (from diagnosis to day before surgery) among women that received surgical treatment after a breast diagnostic workup, a population-based cohort study during 2005-2014 in Skåne, Sweden.

## Data Availability

The data that support the findings of this study are available on request from the corresponding author (F.F.). The data are not publicly available due to Swedish restrictions.

## Abbreviations

CI: Confidence interval
ICD: International Classification of Diseases
IR: Incidence rate
IRR: Incidence rate ratio
SHR: Skåne Healthcare Register
