# Supplementary material for: Psychiatric disorders and cardiovascular diseases during the diagnostic workup of potential breast cancer: a population-based cohort study in Skåne, Sweden
Source: Breast Cancer Res. 2019 Dec 10;21:139. doi: 10.1186/s13058-019-1232-y (PMC6902560; doi:10.1186/s13058-019-1232-y)
Supplement: Supplementary file 1 — Additional file 1: Table S1. ICD codes for breast disorders, psychiatric disorders, and cardiovascular diseases. Figure S1. Study flow chart, a population-based cohort study during 2005-2014 in Skåne, Sweden. Figure S2. Weekly frequency of healthcare visits before and after the date of diagnosis from women with breast cancer, a population-based cohort study during 2005-2014 in Skåne, Sweden. Table S2. Characteristics of women with a breast diagnostic workup by their final diagnosis, a population-based cohort study during 2009-2014 in Skåne, Sweden. Table S3. Incidence rates (IRs, per 1000 person-months) and incidence rate ratios (IRRs) of psychiatric disorders and cardiovascular diseases during the six weeks before diagnosis of women that had a breast diagnostic workup, according to age, calendar period, cohabitation status, and preexisting psychiatric disorder or cardiovascular disease, a population-based cohort study during 2005-2014 in Skåne, Sweden. Figure S3. Incidence rate ratios and their 95% confidence intervals of psychiatric disorders and cardiovascular diseases during the six weeks before diagnosis of women that had a breast diagnostic workup, by type of healthcare visit, a population-based cohort study during 2005-2014 in Skåne, Sweden.*. Table S4. Incidence rates (IRs, per 1000 person-months) and incidence rate ratios (IRRs) of psychiatric disorders and cardiovascular diseases during the waiting time for surgical treatment (from diagnosis to day before surgery) among women that received surgical treatment after a breast diagnostic workup, a population-based cohort study during 2005-2014 in Skåne, Sweden. [file 13058_2019_1232_MOESM1_ESM.docx]

**Supplementary appendix**

**Table S1: ICD codes for breast disorders, psychiatric disorders, and cardiovascular diseases.**

| **Unspecified lump in breast** | N63 |
| --- | --- |
| **Benign breast tumor** | D24, D05 |
| **Breast cancer** | C50 |
| **Psychiatric disorders** | F10-F99 |
| - Stress reaction or adjustment disorder | F43 |
| - Depression | F32-F33 |
| - Anxiety | F40-F41 |
| - Substance abuse | F10-F19 |
| **Cardiovascular diseases** | I00-I99 |
| - Myocardial infarction | I21, I22, I23, I24 |
| - Hypertensive diseases or aneurysm of the heart | I10, I11, I12, I13, I71, I72 |
| - Embolism or thrombosis | I26, I74, I81, I82 |
| - Stroke | I60-I64 |

**Figure S1. Study flow chart, a population-based cohort study during 2005-2014 in Skåne, Sweden.**

Study base: 608,140 adult women living in Skåne during 2005-2014

Skåne Healthcare Register

Swedish Cancer Register

Women without diagnostic workup of potential breast cancer (n=579,479)

Women that had diagnostic workup of potential breast cancer:

Women with breast cancer (n=8,512)

Women with benign tumor (n=4,435)

Women with unspecified lump in breast (n=15,714)

**Figure S2. Weekly frequency of healthcare visits before and after the date of diagnosis from women with breast cancer, a population-based cohort study during 2005-2014 in Skåne, Sweden.**

**
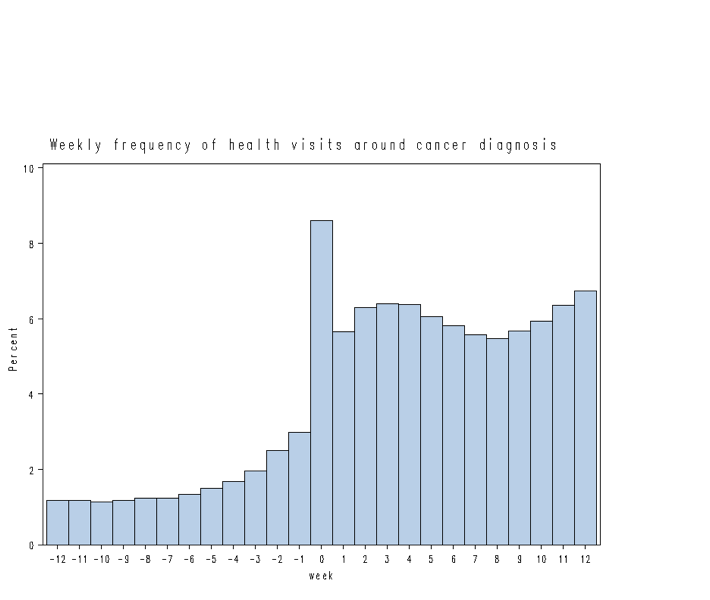
**

Table S2. Characteristics of women with a breast diagnostic workup by their final diagnosis, a population-based cohort study during 2009-2014 in Skåne, Sweden.

| Characteristics | Unspecified lump in breast | Benign tumor | Breast cancer |  |  |  |
| --- | --- | --- | --- | --- | --- | --- |
| No. of women | 10,576 | 2,712 | 5,286 |  |  |  |
| Age in years at diagnosis, mean (SD) | 41 (15) | 46 (16) | 64 (14) |  |  |  |
| Cohabitation status |  |  |  |  |  |  |
| - Cohabitating | 4,868 (46.0%) | 1,297 (47.8%) | 2,707 (51.2%) |  |  |  |
| - Non-cohabitating | 5,708 (54.0%) | 1,415 (52.2%) | 2,579 (48.8%) |  |  |  |
| Reason for workup |  |  |  |  |  |  |
| Due to screening | 697 (6.6%) | 951 (35.0%) | 2,450 (46.4%) |  |  |  |
| Due to symptom | 9,879 (93.4%) | 1,761 65.0%) | 2,836 (53.6%) |  |  |  |
| Tumor stage for screening-detected cancers |  |  |  |  |  |  |
| T0N0M0 / stage 0 / stage I |  |  | 1,820 (74.3%) |  |  |  |
| Stage II |  |  | 540 (22.0%) |  |  |  |
| Stages III & IV | | | |  |  | 75 (3.1%) |
| Missing stage |  |  | 15 (0.6%) |  |  |  |
| Tumor stage for cancers detected due to symptoms |  |  |  |  |  |  |
| T0N0M0 / stage 0 / stage I |  |  | 1,166 (41.1%) |  |  |  |
| Stage II |  |  | 1,240 (43.7%) |  |  |  |
| Stages III & IV | | | |  |  | 377 (13.3%) |
| Missing stage |  |  | 53 (1.9%) |  |  |  |
| Surgical treatment |  |  |  |  |  |  |
| - Yes | 162 (1.5%) | 931 (34.3%) | 4,717 (89.2%) |  |  |  |
| - No | 10,413 (98.5%) | 1,781 (65.7%) | 455 (10.8%) |  |  |  |

**Table S3. Incidence rates (IRs, per 1000 person-months) and incidence rate ratios (IRRs) of psychiatric disorders and cardiovascular diseases during the six weeks before diagnosis of women that had a breast diagnostic workup, according to age, calendar period, civil status, and preexisting psychiatric disorder or cardiovascular disease, a population-based cohort study during 2005-2014 in Skåne, Sweden.**

|  | Reference group* | | | Unspecified lump in breast | | | Benign tumor | | | Breast cancer | | |
| --- | --- | --- | --- | --- | --- | --- | --- | --- | --- | --- | --- | --- |
|  | **No.** | **Crude IR** | **IRR (95% CI)**† | **No.** | **Crude IR** | **IRR (95% CI)**† | **No.** | **Crude IR** | **IRR (95% CI)**† | **No.** | **Crude IR** | **IRR (95% CI)**† |
| Psychiatric disorder | | | | | | | | | | | | |
| Age, years |  |  |  |  |  |  |  |  |  |  |  |  |
| 18-29 | 287,378 | 18.4 | 1.0 | 138 | 25.8 | 1.2 (1.0-1.5) | 21 | 18.8 | 1.1 (0.7-1.6) | 0 | 0.0 | - |
| 30-39 | 189,877 | 20.3 | 1.0 | 181 | 28.5 | 1.1 (1.0-1.3) | 39 | 33.6 | 1.5 (1.1-2.0) | 12 | 29.2 | 1.4 (0.7-2.8) |
| 40-49 | 174,018 | 20.7 | 1.0 | 136 | 26.8 | 1.0 (0.8-1.2) | 38 | 22.5 | 1.0 (0.7-1.3) | 47 | 28.2 | 1.2 (0.9-1.6) |
| 50-59 | 138,529 | 16.6 | 1.0 | 64 | 27.5 | 1.0 (0.7-1.2) | 35 | 36.8 | 1.5 (1.0-2.2) | 49 | 20.8 | 1.0 (0.7-1.4) |
| 60-74 | 113,438 | 13.5 | 1.0 | 46 | 23.3 | 1.1 (0.8-1.5) | 18 | 18.0 | 1.2 (0.8-1.9) | 100 | 20.7 | 1.4 (1.1-1.7) |
| ≥75 | 51,105 | 12.1 | 1.0 | 13 | 16.0 | 0.9 (0.5-1.6) | 4 | 17.7 | 1.1 (0.4-2.7) | 70 | 30.9 | 2.2 (1.7-2.8) |
| Calendar period | |  |  |  |  |  |  |  |  |  |  |  |
| 2005-2009 | 478,678 | 10.1 | 1.0 | 182 | 19.3 | 1.4 (1.2-1.7) | 60 | 20.9 | 1.9 (1.4-2.4) | 91 | 16.7 | 1.7 (1.4-2.1) |
| 2010-2014 | 475,667 | 69.0 | 1.0 | 396 | 31.8 | 0.8 (0.7-0.9) | 95 | 29.0 | 0.9 (0.7-1.0) | 187 | 30.5 | 1.0 (0.9-1.2) |
| Cohabitation status |  |  |  |  |  |  |  |  |  |  |  |  |
| Cohabitating | 318,539 | 13.9 | 1.0 | 195 | 19.2 | 1.0 (0.9-1.2) | 55 | 18.8 | 1.2 (0.9-1.6) | 100 | 16.7 | 1.2 (1.0-1.5) |
| Non-cohabitating | 635,806 | 20.2 | 1.0 | 383 | 32.7 | 1.2 (1.1-1.3) | 100 | 31.0 | 1.3 (1.1-1.6) | 178 | 31.9 | 1.5 (1.3-1.7) |
| Preexisting psychiatric disorder | | | |  |  |  |  |  |  |  |  |  |
| Yes | 805,910 | 71.0 | 1.0 | 500 | 80.1 | 1.1 (1.0-1.2) | 123 | 83.5 | 1.2 (1.0-1.4) | 221 | 84.6 | 1.3 (1.1-1.5) |
| No | 148,435 | 3.5 | 1.0 | 78 | 5.0 | 1.5 (1.2-1.8) | 32 | 6.8 | 2.0 (1.4-2.9) | 57 | 6.4 | 1.8 (1.4-2.4) |
| Cardiovascular Disease | | | | | | | | | | | | |
| Age, years |  |  |  |  |  |  |  |  |  |  |  |  |
| 18-29 | 29,247 | 1.9 | 1.0 | 7 | 1.3 | 0.7 (0.3-1.4) | 2 | 1.8 | 1.1 (0.3-4.4) | 1 | 20.3 | 10.5 (1.3-82.1) |
| 30-39 | 42,457 | 4.5 | 1.0 | 23 | 3.6 | 0.9 (0.6-1.4) | 3 | 2.6 | 0.7 (0.2-2.0) | 2 | 4.9 | 1.1 (0.3-4.2) |
| 40-49 | 89,783 | 10.7 | 1.0 | 43 | 8.5 | 1.0 (0.7-1.3) | 12 | 7.1 | 1.0 (0.6-1.7) | 18 | 10.8 | 1.4 (0.9-2.2) |
| 50-59 | 187,626 | 22.5 | 1.0 | 51 | 21.9 | 1.1 (0.9-1.5) | 17 | 17.9 | 1.1 (0.7-1.8) | 50 | 21.3 | 1.3 (1.0-1.7) |
| 60-74 | 405,993 | 48.2 | 1.0 | 99 | 50.1 | 1.2 (1.0-1.4) | 54 | 53.9 | 1.4 (1.1-1.8) | 301 | 62.2 | 1.6 (1.5-1.8) |
| ≥75 | 339,220 | 80.6 | 1.0 | 74 | 91.0 | 1.0 (0.8-1.3) | 28 | 123.6 | 1.5 (1.0-2.2) | 405 | 179.7 | 2.2 (2.0-2.4) |
| Calendar period |  |  |  |  |  |  |  |  |  |  |  |  |
| 2005-2009 | 598,073 | 12.8 | 1.0 | 118 | 12.5 | 1.4 (1.1-1.6) | 43 | 15.0 | 1.5 (1.1-2.1) | 279 | 51.3 | 1.9 (1.7-2.2) |
| 2010-2014 | 496,253 | 65.2 | 1.0 | 179 | 14.4 | 0.8 (0.7-0.9) | 73 | 22.2 | 1.1 (0.9-1.3) | 498 | 81.3 | 1.7 (1.6-1.9) |
| Cohabitation status |  |  |  |  |  |  |  |  |  |  |  |  |
| Cohabitating | 520,183 | 22.7 | 1.0 | 151 | 14.8 | 1.1 (0.9-1.3) | 49 | 16.8 | 1.1 (0.8-1.4) | 291 | 48.6 | 1.6 (1.4-1.8) |
| Non-cohabitating | 574,143 | 18.2 | 1.0 | 146 | 12.5 | 1.1 (0.9-1.2) | 67 | 20.7 | 1.6 (1.3-2.0) | 486 | 87.1 | 2.1 (2.0-2.3) |
| Preexisting cardiovascular disease | | | |  |  |  |  |  |  |  |  |  |
| Yes | 947,447 | 77.3 | 1.0 | 247 | 58.9 | 1.0 (0.9-1.2) | 95 | 79.5 | 1.3 (1.0-1.6) | 672 | 152.9 | 1.8 (1.7-2.0) |
| No | 146,879 | 3.5 | 1.0 | 50 | 2.8 | 1.3 (1.0-1.7) | 21 | 4.2 | 1.5 (1.0-2.3) | 105 | 14.6 | 2.4 (2.0-2.9) |

Abbreviations: IR, incidence rate; IRR, incidence rate ratio.

*Reference group included person-time accumulated from women who did not have any breast diagnostic workup during the follow-up and the person-time accumulated before the start of workup from women with a breast diagnostic workup during the follow-up.

†CI, confidence interval. IRRs were estimated using Poisson regression, by using attained age as the underlying timescale and adjusting for cohabitation status, registered parish as a proxy for socioeconomic status, and preexisting psychiatric disorders (for outcome of psychiatric disorders) or preexisting cardiovascular diseases (for outcome of cardiovascular diseases).

**Figure S3. Incidence rate ratios and their 95% confidence intervals of psychiatric disorders and cardiovascular diseases during the six weeks before diagnosis of women that had a breast diagnostic workup, by type of healthcare visit, a population-based cohort study during 2005-2014 in Skåne, Sweden.***

*Reference group included person-time accumulated from women who did not have any breast diagnostic workup during the follow-up and the person-time accumulated before the start of workup from women with a breast diagnostic workup during the follow-up. IRRs were estimated using Poisson regression, by using attained age as the underlying timescale and adjusting for cohabitation status, registered parish as a proxy for socioeconomic status, and preexisting psychiatric disorders (for outcome of psychiatric disorders) or preexisting cardiovascular diseases (for outcome of cardiovascular diseases).


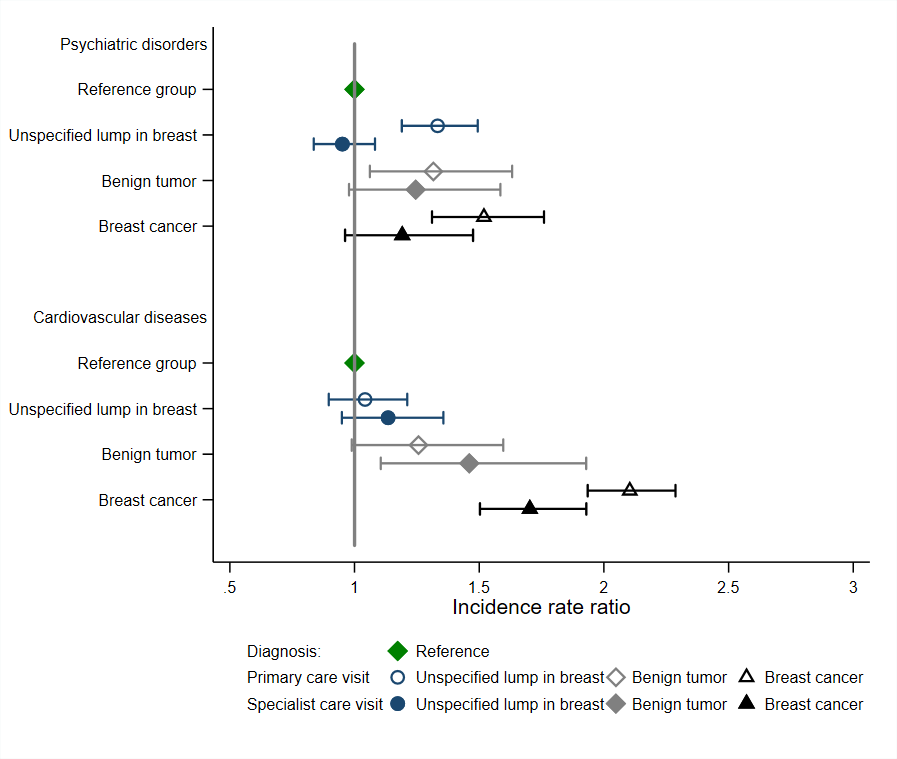


**Table S4. Incidence rates (IRs, per 1000 person-months) and incidence rate ratios (IRRs) of psychiatric disorders and cardiovascular diseases during the waiting time for surgical treatment (from diagnosis to day before surgery) among women that received surgical treatment after a breast diagnostic workup, a population-based cohort study during 2005-2014 in Skåne, Sweden.**

|  | No.† | Crude IR | IRR (95% CI)‡ |
| --- | --- | --- | --- |
| Psychiatric disorder |  |  |  |
| Reference group* | 954,351 | 17.6 | 1.0 |
| Unspecified lump in breast | 10 | 34.3 | 1.8 (0.8-4.2) |
| Benign tumor | 18 | 19.9 | 1.0 (0.7-1.6) |
| Breast cancer | 339 | 29.9 | 1.6 (1.4-1.8) |
| Cardiovascular disease |  |  |  |
| Reference group* | 1,094,327 | 20.1 | 1.0 |
| Unspecified lump in breast | 4 | 13.7 | 1.0 (0.4-2.5) |
| Benign tumor | 22 | 24.3 | 1.1 (0.7-1.7) |
| Breast cancer | 590 | 52.0 | 1.4 (1.3-1.6) |

Abbreviations: IR, incidence rate; IRR, incidence rate ratio.

*Reference group included person-time accumulated from women who did not have any breast diagnostic workup during the follow-up and the person-time accumulated before the start of workup from women with a breast diagnostic workup during the follow-up.

†There were 10 women with unspecified lump in breast (3.4% of all women with lump), 18 women with benign tumor (1.3% of all women with benign tumor) and 339 women with breast cancer (4.2% of all women with breast cancer) that had psychiatric disorders during the waiting time for surgical treatment. There were 4 women with unspecified lump in breast (1.4% of all women with lump), 22 women with benign tumor (1.6% of all women with benign tumor), and 590 women with breast cancer (7.2% of all women with breast cancer) that had cardiovascular diseases during the waiting time for surgical treatment.

‡CI, confidence interval. IRRs were estimated using Poisson regression, by using attained age as the underlying timescale and adjusting for cohabitation status, registered parish as a proxy for socioeconomic status, and preexisting psychiatric disorders (for outcome of psychiatric disorders) or preexisting cardiovascular diseases (for outcome of cardiovascular diseases).
